# Low rates of prior HIV testing among HIV-positive adults accessing outpatient services in Eswatini

**DOI:** 10.1186/s12981-019-0254-y

**Published:** 2019-12-05

**Authors:** Harriet Nuwagaba-Biribonwoha, Yingfeng Wu, Averie Baird Gachuhi, Margaret L. McNairy, Veli Madau, Mathew Lamb, Sikhathele Mazibuko, Zandile Mnisi, Sean Burke, Neena Philip, Ruben Sahabo, Wafaa M. El Sadr

**Affiliations:** 1 ICAP at Columbia University, Mailman School of Public Health, Mbabane, Eswatini; 2 Department of Epidemiology, Mailman School of Public Health, New York, NY USA; 3 ICAP at Columbia University, Mailman School of Public Health, New York, NY USA; 4 Weill Cornell Medical College, New York, NY USA; 5 Ministry of Health, Mbabane, Eswatini

**Keywords:** HIV testing, Awareness of HIV status, First 90, First 95, Eswatini

## Abstract

Prior HIV testing and awareness of HIV-positive status were assessed among HIV-positive adults at 20 clinics in Eswatini. Of 2196 HIV-positive adults, 1183 (53.8%) reported no prior HIV testing, and 1948 (88.7%) were unaware of their HIV-positive status. Males [adjusted odds ratio, AOR, (95% confidence interval): 0.7 (0.5–0.9)], youth 18–25 years [AOR 0.6 (0.4–0.95)], adults ≥ 50 years [AOR 0.5 (0.3–0.9)], those needing family support [AOR 0.6 (0.5–0.8)], and those living ≥ 45 min from clinic [AOR 0.5 (0.4–0.8)] were less likely to know their HIV-positive status. More HIV testing is needed to achieve 95-95-95 targets, with targeted strategies for those less likely to test for HIV.

## Introduction

HIV testing is the gateway to HIV prevention and treatment services. Individuals who test HIV-negative can then access HIV prevention services, while those who test HIV-positive access antiretroviral therapy (ART). Universal access to HIV testing services (HTS) is a high priority in the Kingdom of Eswatini (formerly Swaziland), a country with a severe generalised HIV epidemic [1]. In order to achieve HIV epidemic control, Eswatini, like other sub-Saharan African countries, has committed to the UNAIDS 95:95:95 targets: by 2030, 95% of people living with HIV (PLHIV) should be diagnosed, 95% of those diagnosed should be on treatment and 95% of those on treatment should be virally suppressed [2].

There has been significant investment in HTS in Eswatini in order to achieve 95-95-95 targets. National program data indicate that over 400,000 HIV tests are conducted annually in this country which has a population of 1.1 million people [3]. Data from two sequential nationally-representative population surveys show that at population level, awareness of HIV positive status has increased from 62% among adults 18–49 years in 2011 [4] to 74% among adults 15–49 years in 2016/2017 [1]. While these findings are encouraging, a substantial testing gap remains with disproportionately low HIV testing rates among sub-groups of the population, which in turn compromises achievement of the first 95 target. To explore the rates of HIV testing and previous knowledge of HIV-positive status, we assessed previous HIV testing history among adults attending outpatient clinics in Eswatini.

## Methods

We analysed baseline HIV testing data from the Link4Health, a cluster-randomized implementation science study conducted at 20 health facilities in Eswatini from all 4 regions of Eswatini [5, 6]. Participating health facilities included clinics paired with hospitals, matched by clinic size implementing partner and urban/rural location before randomization. The Link4Health study assessed linkage to care after HIV diagnosis, as well as retention in care and viral load suppression after 1 year of follow-up. Adults ≥ 18 years, testing HIV-positive and self-reporting as not having been enrolled in HIV care in the prior six months were eligible for the Link4Health study. The Link4Health study recruited participants between August 2013 and November 2014.

We analysed baseline study data of the HIV-positive participants enrolled in the study for (1) history of prior HIV testing, i.e. self-report of a prior HIV test, irrespective of the HIV test result, as well as reasons for ever or never testing; and (2) self-reported awareness of HIV-positive status prior to the study baseline HIV-positive test. In the latter group of participants, adults unaware of their HIV-positive status included those who had never tested previously, those with a previous HIV-negative test, and those with a previous indeterminate or unknown test result. Hierarchical logistic regression models were used to generate unadjusted and adjusted odds ratios [AOR, 95% Confidence Intervals] for the above two outcomes, controlling for available demographic, household and behavioural characteristics. Clinic-level cluster effects were controlled with clinic random effects and bivariate analyses with p < 0.2 entered into the final multivariable model.

## Results

Of 2196 HIV-positive participants [59% female, median age 31 years, IQR 26–39], 1181 [53.8%] reported no prior HIV test. Correlates of no prior HIV testing are presented in Table 1. The odds of no prior HIV testing were higher among males compared to females [AOR 1.7, 1.4–2.0]. Using the 40–49 years age group as a reference, adults 50 years and older had higher odds of no prior HIV testing [AOR 1.9, 1.3–2.9], but no statistically significant differences were observed with other age-groups. Staying at home showed a borderline association with no prior HIV testing [AOR 1.3, 1.0–1.7], in comparison to adults who had travelled away from home in the previous 12 months. Additionally, adults who indicated the need for more family support had higher odds of no prior HIV test compared to those who reported needing the same or less family support [AOR 1.5, 1.2–1.8]. One-way travel time from home to the clinic exceeding 45 min also had a borderline association with no prior HIV testing [AOR 1.2, 1.0–1.5], in comparison to adults who travelled for a shorter time.

**Table 1:** Correlates of no prior HIV testing and awareness of HIV-positive status among newly identified people living with HIV (PLHIV) in the Link4Health study

	Total, N (%)	Correlates of no prior HIV testing among newly identified PLHIV participating in Link4Health				Correlates of awareness of HIV-positive status among newly identified PLHIV participating in Link4Health			
		Never had an HIV test, n (%)	Ever had an HIV test, n (%)	Crude OR (95% CI) for never tested vs ever tested	Adjusted OR (95% CI) for never tested vs ever tested	Aware of HIV-positive status, n (%)	Not aware of HIV-positive status^a^ (%)	Crude OR (95% CI) for aware versus not aware	Adjusted OR (95% CI) for aware versus not aware
Total	2196 (100)	1181 (53.8)	1015 (46.2)	–	–	248 (11.3)	1948 (88.7)	–	–
Sex									
Female	1290 (58.7)	629 (53.3)	661 (65.1)	1.0	1.0	166 (66.9)	1124 (57.7)	1.0	1.0
Male	906 (41.3)	552 (46.7)	354 (34.9)	1.7 (1.4,2.0)	1.7 (1.4, 2.0)	82 (33.1)	824 (42.3)	0.7 (0.5, 0.9)	0.7 (0.5, 0.9)
Age									
18–25 years	524 (23.9)	244 (20.7)	280 (27.6)	0.6 (0.5, 0.8)	0.8 (0.6, 1.1)	51 (20.6)	473 (24.3)	0.7 (0.4, 1.1)	0.6 (0.4, 0.95)
26–30 years	540 (24.6)	261 (22.1)	279 (27.5)	0.7 (0.5, 0.9)	0.8 (0.6, 1.0)	71 (28.6)	469 (24.1)	1.0 (0.6, 1.5)	0.9 (0.6, 1.4)
31–39 years	596 (27.1)	334 (28.3)	262 (25.8)	0.9 (0.7, 1.2)	1.0 (0.7, 1.3)	72 (29.0)	524 (26.9)	0.9 (0.6, 1.4)	0.9 (0.6, 1.3)
40–49 years	324 (14.8)	187 (15.8)	137 (13.5)	1.0	1.0	41 (16.5)	283 (14.5)	1.0	1.0
50+ years	212 (9.7)	155 (13.1)	57 (5.6)	2.0 (1.4, 2.9)	1.9 (1.3, 2.9)	13 (5.2)	199 (10.2)	0.4 (0.2, 0.8)	0.5 (0.3, 0.9)
Was away from home during the last 12 months									
Stayed at home	1840 (84)	1015 (86.3)	825 (81.4)	1.4 (1.1, 1.8)	1.3 (1.0, 1.7)	198 (79.8)	1642 (84.6)	0.8 (0.5, 1.1)	–
Away 1 or more times	349 (16)	161 (13.7)	188 (18.6)	1.0	1.0	50 (20.2)	299 (15.4)	1.0	–
Family support needed									
More	1275 (60.2)	755 (65.5)	520 (53.9)	1.6 (1.4, 2.0)	1.5 (1.2, 1.8)	115 (48.7)	1160 (61.6)	0.6 (0.4, 0.8)	0.6 (0.5, 0.8)
Same or less	843 (39.8)	398 (34.5)	445 (46.1)	1.0	1.0	121 (14.4)	722 (38.4)	1.0	1.0
Travel time from home to this clinic (one way)									
< 45 min	1411 (64.3)	747 (63.3)	664 (65.4)	1.0	1.0	179 (72.2)	1232 (63.2)	1.0	1.0
≥ 45 min	785 (35.8)	434 (36.8)	351 (34.6)	1.3 (1.1, 1.6)	1.2 (1.0, 1.5)	69 (27.8)	716 (36.8)	0.6 (0.4, 0.8)	0.5 (0.4, 0.8)
Someone else in the household has HIV									
Yes	775 (37.7)	426 (38.2)	349 (37.1)	1.2 (0.9, 1.6)	–	91 (39.9)	684 (37.4)	1.0	–
No	1281 (62.3)	689 (61.8)	592 (62.9)	1.0	–	137 (60.1)	1144 (62.6)	0.9 (0.6, 1.2)	–
HIV knowledge (Cronbach alpha = 0.59)									
Low	872 (39.7)	493 (41.7)	379 (37.3)	1.2 (1.0, 1.4)	–	97 (39.1)	775 (39.8)	0.9 (0.7, 1.2)	–
High	1324 (60.3)	688 (58.3)	636 (62.7)	1.0	–	151 (60.9)	1173 (60.2)	1.0	–

Not aware of HIV-positive status included adults never tested before, and those whose pervious HIV test result was HIV negative, indeterminate or unknown

Among adults with no prior HIV testing, the most common reasons for never testing were feeling sick [61%] and being worried about HIV [35%]. These were also the most common reasons for testing for HIV among adults who had tested for HIV previously: feeling sick [58%], and being worried about HIV [34%].

Among all participants, of 2196, the majority were unaware of their HIV-positive status [1948, 88.7%], with only 248 [11.3%] adults aware that they were living with HIV. Males were less likely to be previously aware of their HIV-positive status compared to females [AOR 0.7, 0.5–0.9]. Youth 18–25 years [AOR 0.6, 0.4–0.95] and adults 50 years and older [AOR 0.5, 0.3–0.9] were less likely to be aware of their HIV positive status, compared to adults 40–49 years. Additionally, adults who reported needing more family support [AOR 0.6, 0.5–0.8] and those who lived 45 or more minutes away from the HIV clinic (one way travel) [AOR 0.5, 0.4–0.8] were less likely to be aware of their HIV-positive status.

## Discussion

Over half of HIV-positive adults enrolled in our study reported no history of prior HIV testing, and only 1 in 10 were aware of their HIV-positive status, demonstrating a substantial HIV testing gap. We observed significantly lower awareness of HIV-positive status than reported in prior national population-based household surveys [4, 7], suggesting existence of sub-populations underserved with HIV testing services in Eswatini. Prior HIV testing was lower among men, a group that has persistently lagged in accessing testing services in Eswatini [4, 7] and globally [8]. Testing was also lower among those who reported no travel in the previous 12 months, which may be an indication of lack of access to testing services within communities where these participants reside. Recent efforts to scale-up HIV self-testing and community testing could potentially mitigate this gap.

The low prevalence of prior testing among older adults may be due to the lack of HIV testing services specifically tailored to the needs of older individuals and a possible misconception that older individuals may not be at risk for HIV. Our study suggests the need for interventions to engage older adults in HIV testing. We also noted lower prior HIV testing among HIV-positive participants who reported needing more family support, which could be due to stigma experienced by such individuals. Continued social support is needed to encourage individuals to access HIV testing services. Our study showed that the most common reasons for never testing for HIV and for testing for HIV were similar, i.e. being ill and being concerned about HIV, further emphasizing the need for appropriate messages, counselling and social support to address concerns related to HIV testing.

We found HIV positive males, youth, older adults, those needing more family support and those living further from clinics were less likely to be aware of their HIV-positive status. These individuals are likely unknowingly transmitting HIV to others, if HIV infection is undiagnosed, untreated and viral load unsuppressed. Our findings suggest that demand generation efforts for HTS should be focused on specific populations. Emphasis should be placed on knowledge of HIV positive status as the gateway to treatment, which can be life-saving for the individual and protective of transmission to others, and knowledge of HIV negative status as the gateway to prevention services, including pre-exposure prophylaxis and voluntary medical male circumcision for men [9]. In addition to facility-based testing, targeted community approaches are needed to enhance knowledge of status. Innovations such as HIV self-testing, partner notification and index case testing, as well as community testing venues [10–12] should be considered.

Our study’s strengths were inclusion of a large number of health facilities (both hospitals and clinics) located throughout the country, and availability of data on history and motivation for HIV testing. However, data on prior HIV testing and knowledge of HIV-positive status were self-reported, and there may have been reporting bias as adults already knew they were HIV positive by the time of the interview. Nonetheless, we demonstrated significant gaps in prior HIV testing and the first 95, which require intensified efforts in clinic and public health programs offering HIV testing services.

## Conclusion

Over half of the HIV-positive persons in this study had no prior HIV testing and only 1 in 10 of the participants were previously aware of their HIV-positive status. In this setting of high HIV prevalence and incidence, these low testing rates can compromise achievement of the 95-95-95 targets. Males, youth, older adults, those needing more family support and those living further from clinics are priority populations for tailored strategies to enhance their access to HIV testing services. These findings indicate the need for further strengthening of HIV testing services, with attention to sub-populations that tend to lag behind in HIV testing.

## Data Availability

Not publically available.
